# Effects of L1-ORF2 fragments on green fluorescent protein gene expression

**DOI:** 10.1590/S1415-47572009005000068

**Published:** 2009-12-01

**Authors:** Xiu-Fang Wang, Xia Jin, Xiaoyan Wang, Jing Liu, Jingjing Feng, QinQing Yang, Wenli Mu, Xiaojuan Shi, Zhanjun Lu

**Affiliations:** Hebei Key Lab of Laboratory Animal, Department of Genetics, Hebei Medical University, Shijiazhuang, Hebei ProvinceChina

**Keywords:** gene expression, green fluorescent protein gene, L1-ORF2, transcription termination, orientation

## Abstract

The retrotransposon known as long interspersed nuclear element-1 (L1) is 6 kb long, although most L1s in mammalian and other eukaryotic cells are truncated. L1 contains two open reading frames, ORF1 and ORF2, that code for an RNA-binding protein and a protein with endonuclease and reverse transcriptase activities, respectively. In this work, we examined the effects of full length L1-ORF2 and ORF2 fragments on green fluorescent protein gene (*GFP*) expression when inserted into the pEGFP-C1 vector downstream of *GFP*. All of the ORF2 fragments in sense orientation inhibited *GFP* expression more than when in antisense orientation, which suggests that small ORF2 fragments contribute to the distinct inhibitory effects of this ORF on gene expression. These results provide the first evidence that different 280-bp fragments have distinct effects on the termination of gene transcription, and that when inserted in the antisense direction, fragment 280-9 (the 3' end fragment of ORF2) induces premature termination of transcription that is consistent with the effect of ORF2.

## Introduction

Type 1 long interspersed nuclear elements (L1s) are the most abundant autonomous retrotransposons in mammals, and comprise 17% of the human genome (Kazazian and Moran, 1998; Smit *et al.*, 1999; Lander *et al.*, 2001; Abrusán *et al.*, 2008). Intact L1 is ~6 kb long, has an internal promoter for RNA polymerase II and encodes two polypeptides essential for retrotransposition (Swergold, 1990; Moran *et al.*, 1996; Athanikar *et al.*, 2004). The product of ORF1 is an RNA-binding protein, whereas ORF2 encodes a protein with endonuclease and reverse transcriptase activities (Feng *et al.*, 1996; Martin and Bushman, 2001; Cost *et al.*, 2002; Weichenrieder *et al.*, 2004; Martin *et al.*, 2005). L1 elements replicate via target-site primed reverse transcription, which combines chromosomal insertion with reverse transcription (Cost *et al.*, 2002).

Although elements of L1 can occur almost anywhere in the mammalian genome, their abundance varies among genomic regions. In general, these elements are much more abundant in genomic regions that are AT-rich, have a low-recombination frequency and are gene-poor (Pavlicek *et al.*, 2001; Yang *et al.*, 2004; Hackenberg *et al.*, 2005; Belancio *et al.*, 2006; Graham and Boissinot, 2006). In human genes, L1s preferentially have an antisense orientation and most copies are truncated (Sassaman *et al.*, 1997; Boissinot *et al.*, 2000; Sheen *et al.*, 2000; Lander *et al.*, 2001), rearranged (Skowronski and Singer, 1986) or both. These findings imply that the length and orientation of L1s have different effects on genes. It would therefore seem highly important to study the effects of L1 fragments and their orientations on gene expression.

L1s can cause the retrotransposition of *Alu* (Dewannieux *et al.*, 2003) and mediate the cell growth and differentiation associated with this event (Ergün *et al.*, 2004; Sciamanna *et al.*, 2005). Han *et al.* (2004) reported that L1.2-ORF2 in the sense orientation inhibited *GFP* expression much more than when in antisense orientation. By using appropriate deletions these authors also showed that the inhibition of gene expression varied with the length of the L1.2-ORF2 fragment.

In this study, we used L1PA3, a subfamily of L1s that shares 96% similarity with L1.2-ORF2, to examine whether L1PA3-ORF2 has the same effect on gene expression as L1.2-ORF2. Seven 280-bp fragments obtained by the polymerase chain reaction (PCR) from different regions of L1PA3-ORF2 (ORF2) were fused in tandem to *GFP* in order to examine their effect on gene expression.

## Materials and Methods

###  Plasmid construction

Tandem repeat plasmids (Table 1) were constructed as previously described (Okano *et al.*, 2008) and were identified by digestion with the restriction enzyme pair *Hin*dIII/ *Nhe* I and DNA sequencing (Generay Co. Shanghai, China). The primers used for PCR are shown in Table 2.

###  Cell culture and cell transfection

HeLa cells were routinely cultured in Dulbecco's modified Eagle's medium (DMEM) with 10% fetal calf serum (FCS). Aliquots containing 1.8 x 10^5^ cells/mL were plated in 12-well plates and then cultured at 37 °C in 5% CO_2_ for 24 h. At approximately 50%-70% confluence, the cells were transfected with 1.5 μg of plasmid DNA and 3 μL of liposomes (Lipofectamine 2000; Invitrogen, Grand Island, NY) in order to observe fluorescent cells and to generate RNA for subsequent experiments.

###  Assessment of *GFP* reporter protein

The expression of *GFP* reporter protein was assessed by fluorescence microscopy of transfected HeLa cells.

###  Northern blotting

The *GFP* probe was labeled with [^32^P]-deoxycytidine triphosphate (dCTP) via PCR using the primers shown in Table 2. Total RNA was extracted from plasmid-transfected HeLa cells with Trizol reagent (Invitrogen, Inc.) 36 h after transfection. The RNA was electrophoresed in a 1.2% agarose gel denatured with 3% formaldehyde followed by transferring to nylon membranes in 20x salt-sodium citrate (SSC) for 24 h. RNA was cross-linked to the membranes by exposure to UV light and the membranes then incubated with the *GFP* probe at 42 °C followed by autoradiography. The membranes were subsequently stripped by washing twice at 80 °C for 1 h with 50 mM Tris, pH 7.4, containing 50% formamide and 5% sodium dodecylsulfate (SDS), and then hybridized with a [^32^P]-labeled probe for neo mRNA (the cassette for neomycin resistance). This probe was prepared by PCR amplification with the primers shown in Table 2.

## Results

###  Effects of ORF2 in sense and antisense orientations on *GFP* expression

**Figure 1:**
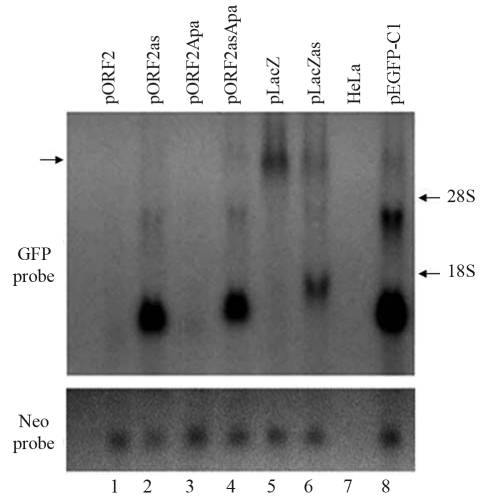
Insertion of ORF2 in different orientations exerted distinct inhibitory effects on gene expression. Total RNA extracted from HeLa cells transfected with plasmids was analyzed by northern blotting. ORF2 in sense orientation inhibited *GFP* expression much more strongly than in antisense orientation; in the latter orientation ORF2 also caused premature transcriptional termination. Arrow on the left shows the expected positions of GFPORF2 and GFPlacZ that are of the same length.

ORF2 (3825 bp) or the *lacZ* sequence was inserted downstream of *GFP* in the pEGFP-C1 vector. The insertion of ORF2 in sense or antisense orientation significantly decreased *GFP* RNA (Figure 1) and protein (data not shown) expression. To demonstrate this decrease, we used *Xho* I /*Pst* I or *Apa* I restriction enzymes to construct plasmids of pORF2, pORF2as, pORF2Apa and pORF2asApa (see Table 1). When ORF2 was inserted in the sense orientation (pORF2), *GFP* RNA production was only 3.6% of that seen with ORF2 in the antisense orientation (pORF2as) (Figure 1, lane 1 *vs.* lane 2), and when ORF2Apa was inserted in the sense orientation (pORF2Apa) *GFP* RNA production was 4.2% of that seen with ORF2Apa in the antisense orientation (pORF2asApa) (Figure 1, lane 3 *vs.* lane 4). Thus, when ORF2 or ORF2Apa was inserted in the antisense orientation most of the decrease in the expression of full-length *GFP* RNA was related to the generation of low molecular mass RNA species, indicating that antisense ORF2 caused premature termination of *GFP* transcription. The insertion of *lacZ* in either orientation reduced RNA synthesis to low similar levels (Figure 1, lanes 5 and 6). The insertion of *lacZ* in antisense orientation caused premature termination of *GFP* transcription (Figure 1, lane 6), whereas the insertion of this gene in sense orientation induced transcriptional elongation (Figure 1, lane 5). These findings indicated that ORF2 in sense orientation caused much stronger gene inhibition than in antisense orientation, with the latter causing premature transcriptional termination.

###  Effects of different 280-bp fragments of ORF2 on *GFP* expression

**Figure 2:**
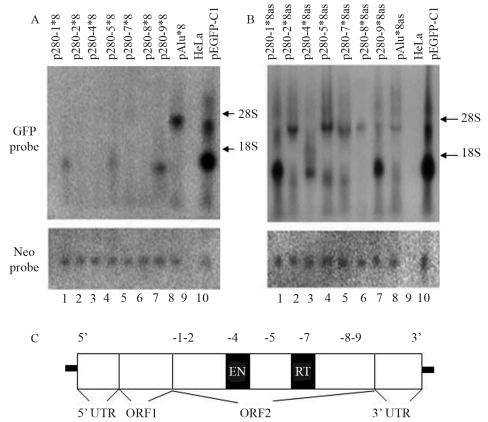
Effects of 280-bp ORF2 fragments on *GFP* transcription. (A) The effects of seven 280-bp ORF2 fragments in sense orientation on gene transcription. Fragments 280-1, 280-5 and 280-9 fragments caused premature transcriptional termination and produced low molecular mass RNA (lanes 1, 4 and 7), whereas fragments 280-2, 280-7 and 280-8 did not cause premature termination of *GFP* transcription. (B) Effects on gene transcription of the same seven 280-bp ORF2 fragments in antisense orientation. Fragments 280-1, 280-4 and 280-9 caused premature transcriptional termination whereas other 280-bp fragments did not. (C) The basic structure of L1 and amplification sites of different 280-bp fragments. An intact L1 consists of 5' UTR, ORF1, ORF2 and 3'UTR. EN: endonuclease; RT: reverse transcriptase. -1~-9 indicates the sites of fragments obtained from ORF2. -1: 280-1 fragment, -2: 280-2 fragment, -4: 280-4 fragment, -5: 280-5 fragment, -7: 280-7 fragment, -8: 280-8 fragment and -9: 280-9 fragment.

To study the effects of ORF2 fragments on *GFP* expression, we obtained seven 280-bp fragments from different regions of ORF2, as shown in Figure 2C and Table 2. Head and tail, tandem 8-sequence repeats (see Table 1) were constructed for each fragment. As shown in Figure 2, all of the inserts inhibited *GFP* transcription much more strongly in sense than in antisense orientation, which was consistent with the results for full-length ORF2 (Figure 1). Regardless of their orientation (sense or antisense), fragments 280-1 and 280-9 caused premature termination of transcription and produced low molecular mass RNA (Figure 2A, lanes 1 and 7; Figure 2B, lanes 1 and 7). Fragment 280-5 caused premature termination of *GFP* transcription in sense orientation (Figure 2A, lane 4), whereas fragment 280-4 had the same effect in antisense orientation (Figure 2B, lane 3). Other ORF2 fragments, including fragments 280-2, 280-7 and 280-8, did not induce premature termination of *GFP* transcription in either orientation. Thus, in contrast to ORF2 which caused premature termination of *GFP* transcription when in antisense orientation, the effect of ORF2 fragments on transcriptional elongation were less predictable.

###  Effects of simple repeats constructed from ORF2 fragments on *GFP* expression

**Figure 3:**
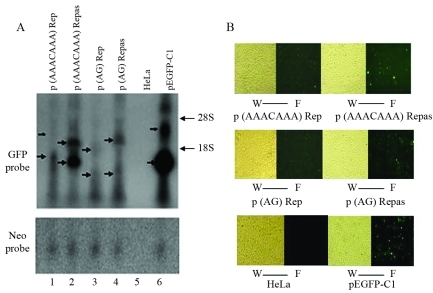
Effects of simple repeats constructed from small A-rich ORF2 fragments on *GFP* gene expression. AAACAAA or AG 736-bp long repeats were inserted in sense or antisense orientation downstream of *GFP*. The inserts inhibited *GFP* transcription. AAACAAA repeats in either orientation induced premature transcriptional termination. AG repeats in antisense orientation produced a greater number of higher molecular mass transcripts than in sense orientation. Arrows indicate positions of low or high molecular mass transcripts.

Since the different 280-bp ORF2 fragments had distinct effects on *GFP* expression in HeLa cells (Figure 2), we examined the influence of even shorter ORF fragments on gene expression. As shown in Table 3, the ORF2 fragments generally contained more A than T. We chose AAACAAA and AG, which are particularly abundant in ORF2, and constructed 736-bp repeats of these base sequences. The AAACAAA or AG repeats were then inserted into the pEGFP-C1 vector downstream of *GFP* in sense or antisense orientation. Fragments inserted in sense orientation suppressed transcription more strongly than those in antisense orientation (Figure 3), in agreement with the findings for ORF2 and its 280-bp fragments. Interestingly, AAACAAA repeats in either orientation caused premature transcriptional termination (Figure 3, lanes 1 and 2), whereas AG repeats in antisense orientation resulted in greater synthesis of higher molecular mass transcripts than did AG repeats in sense orientation (Figure 3, lane 4 *vs.* lane 3).

###  ORF2 fragment 280-9 is responsible for premature transcriptional termination by ORF2 in antisense orientation

**Figure 4:**
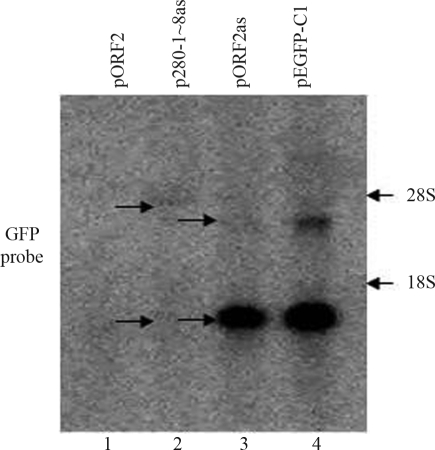
The 280-1~8 fragment (generated by deleting 280-9 and its downstream region in ORF2) did not induce premature transcriptional termination when inserted in antisense orientation downstream of *GFP* (lane 2). Arrows show the positions of low or high molecular mass transcripts.

As shown above (Figure 2), the ORF2 fragments had distinct effects on *GFP* transcriptional elongation. Of seven 280-bp fragments, fragment 280-9 (the 3' end sequence of ORF2) caused premature transcriptional termination when inserted in antisense orientation (Figure 2B), in agreement with the results for ORF2 (Figure 1). These findings implied that when ORF2 is in antisense orientation the 3' end of ORF2 is responsible for premature transcriptional termination. To confirm this, the 3' end of ORF2, including fragment 280-9 and its downstream region, were deleted (the resulting fragment was referred to as 280-1~8). When fragment 280-1~8 was inserted in the antisense orientation downstream of *GFP* there was no premature transcriptional termination of this gene (Figure 4, lane 2), thus confirming the importance of the 3' end sequence of ORF2 in this phenomenon.

### ORF2 280-bp segments cause length-dependent reduction of RNA and protein expression

**Figure 5:**
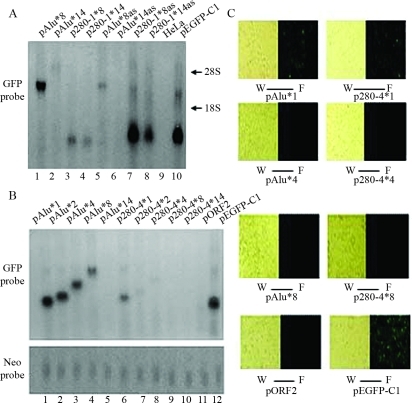
Length-dependent inhibition of *GFP* transcription by fragment 280-1 inserted in either orientation (A) and fragment 280-4 in sense orientation (B), and of protein expression (C). *Alu* was used as a positive control in these experiments.

We inserted 8 or 14 copies of ORF2 fragment 280-1 downstream of *GFP* in the pEGFP-C1 vector. With fragment 280-1 in either the sense or antisense orientation, the construct inhibited gene transcription in a length-dependent manner (Figure 5A, lane 4 *vs.* lane 3; lane 8 *vs.* lane 7). However, insertion of fragment 280-1 in sense orientation induced much stronger inhibition of *GFP* expression than did its insertion in antisense orientation, and when present in either orientation this fragment caused premature transcriptional termination.

Copies of ORF2 fragment 280-4 inserted in sense orientation downstream of *GFP* decreased RNA transcription (Figure 5B) and protein expression (Figure 5C) in a length-dependent manner. The lengths of RNA transcripts increased with increasing numbers of copies of fragment 280-4 (Figure 5B, lanes 6-10), suggesting that fragment 280-4 did not cause premature termination of transcription.

*Alu*, used as a control in these experiments, also inhibited gene expression in a length-dependent manner but did not cause premature transcriptional termination (Figure 5A, lane 2 *vs.* lane 1 and lane 6 *vs.* lane 5; Figure 5B, lanes 1-5).

## Discussion

L1 elements are associated with a number of biological phenomena including X chromosome inactivation (Bailey *et al.*, 2000; Lyon, 2000), monoallelic gene expression (Allen *et al.*, 2003), gene rearrangement (Burwinkel and Kilimann, 1998), tumorigenesis (Martin and Branciforte, 1993) and organic evolution (Deininger *et al.*, 2003; Hedges and Batzer, 2005). Most L1s in the human genome are truncated (Sassaman *et al.*, 1997; Boissinot *et al.*, 2000; Sheen *et al.*, 2000) and L1 sequences found in introns are preferentially located in the antisense orientation (Smit, 1999; Medstrand *et al.*, 2002). These characteristics provide an interesting situation for examining the influence of L1 fragments and their orientation on gene expression.

As shown here, the ORF2 of L1PA3 in sense orientation inhibited *GFP* expression much more than in antisense orientation, and caused premature transcriptional termination in the latter orientation, in agreement with previous findings for L1.2-ORF2 (Han *et al.*, 2004). Although the sequences of L1.2-ORF2 and L1PA3-ORF2 are not identical, they had similar effects on gene expression, suggesting that mutation of individual nucleotides does not affect the functions of this ORF.

Different restriction enzymes were used to construct plasmids with ORF2 in sense (pORF2 and pORF2Apa) and antisense (pORF2as and pORF2asApa) orientation. The insertion of ORF2 (Figure 1, lanes 1 and 2) or ORF2Apa (Figure 1, lanes 3 and 4) had the same effect on gene expression as when they were incorporated into plasmids in the same orientation, a finding that increased our confidence in the results of this study. The ORF2 sequence does not inhibit the initiation of transcription and is a poor substrate for transcriptional elongation (Han *et al.*, 2004).

The influence of ORF2 fragments on transcriptional termination and gene inhibition was examined by using seven 280-bp ORF2 fragments lacking restriction enzyme sites that could otherwise disturb the base linkage within the fragments. Each fragment consisted of eight tandem repeats and was inserted downstream of *GFP* in sense or antisense orientation. All of the fragments significantly reduced gene expression, with greater inhibition when in sense compared to antisense orientation (Figure 2); this finding consistent with our observations for ORF2 (Figure 1).

Enhanced RNA degradation or decreased RNA production could reduce RNA concentrations. Han *et al.* (2004) stated that most of the decrease in *GFP*-ORF2 transcription in the presence of L1.2-ORF2 was not due to transcript degradation. In the present study, the bands seen in northern blots probably reflected the rate of gene transcription.

The 280-bp ORF2 fragments had different effects on transcriptional elongation. Fragments 280-1 and 280-9 in sense or antisense orientation caused premature transcriptional termination (Figure 2A, lanes 1 and 7; Figure 2B, lanes 1 and 7). Fragment 280-5 in sense orientation (Figure 2A, lane 4) and fragment 280-4 in antisense orientation (Figure 2B, lane 3) also caused premature transcription termination. Other 280-bp fragments in either orientation did not cause premature transcription termination.

Mutations involving individual nucleotides did not affect ORF2 function, and each 280-bp fragment continued to have a stronger inhibitory effect in sense compared to antisense orientation. These findings prompted us to investigate the effects of fragments < 280 bp on gene expression. L1 has an adenosine-rich (A-rich) bias in the sense strand (Deininger *et al.*, 2003). We chose small fragments of AAACAAA and AG, which are abundant in ORF2. Fragments containing tandem repeats of AAACAAA and AG were used to ensure a sufficiently large effect on gene expression. As in the experiments with the 280-bp fragments, the AAACAAA and AG repeats showed much stronger inhibition in sense compared to antisense orientation. AAACAAA sequences were certified to be Sox2 protein binding sites. The binding of Sox2 protein to these sites suppresses gene expression driven by L1 5'-UTR (Muotri *et al.*, 2005) and may be one of the mechanisms by which AAACAAA inhibits gene expression.

Figure 2 shows that the 280-bp ORF2 fragments had different effects on transcriptional termination. Fragment 280-9 in antisense orientation caused premature transcriptional termination (Figure 2B, lane 9) that resembled the results obtained with ORF2 in antisense orientation (Figure 1, lane 2). This finding suggested that fragment 280-9 plays a key role in premature transcriptional termination by ORF2. To confirm this hypothesis, we deleted fragment 280-9 from the 3'end of ORF2 and inserted the resulting fragment (280-1~8) in antisense orientation downstream of *GFP*. This insert failed to stimulate the production of low molecular mass RNA similar to that seen with ORF2 in antisense orientation (see Figure 4). This result indicated that fragment 280-9 and its 3'end sequence play a key role in the premature transcriptional termination mediated by ORF2.

Since the chromosomal densities of *Alu* and L1 are negatively correlated with each another (except for the Y chromosome), and since L1 elements are responsible for the retrotransposition of *Alu* retroelements (Dewannieux *et al.*, 2003), *Alu* was used as a parallel control in some experiments. The genomic distribution of *Alu* is suggestive of a possible involvement in enhancing gene expression. However, as shown here, *Alu* inhibited gene expression in a length-dependent manner (Figure 5), but had a much weaker effect than ORF2 fragments 280-1 or 280-4. In addition, *Alu* did not cause premature transcriptional termination. ORF2 may cause premature transcriptional termination (Figure 1, lanes 1-4; Figure 4, lanes 1 and 3) through the presence of multiple functional canonical and noncanonical polyA signals in L1 (Deininger *et al.*, 2003). Such signals are also present in some ORF2 fragments, *e.g.*, fragments 280-9 and 280-1, where they presumably also promote termination. Han *et al.* (2004) found that tandem L1.2-ORF1 caused length-dependent inhibited of gene expression. As shown here, 280-1, 280-4 and *Alu* in either orientation also caused length-dependent suppression of gene expression.

In conclusion, we have described a number of potentially important functions of ORF2 and its fragments that affect gene expression. The major findings of this work are that: (1) ORF2 fragments contributed differently to gene transcriptional elongation, with only some fragments inducing the premature transcriptional termination seen with ORF2, (2) in deletion studies, the 3' end sequence of ORF2 (fragment 280-9) is responsible for the premature transcriptional termination observed with ORF2 in antisense orientation, and (3) all of the ORF2 fragments studied here, as well as ORF2 itself, inhibited gene expression much more in sense compared to antisense orientation. The latter observation suggested that small fragments contributed to ORF2-mediated inhibition of gene expression primarily when in sense orientation.

## Figures and Tables

**Table 1:** Plasmids used in this study.

Plasmids	Fragment inserted into pEGFP-C1 and annotation
pORF2, pORF2as	ORF2 inserted in sense or antisense (as) orientation downstream of *GFP* by using the restriction enzymes *Xho* I /*Pst* I.
pORF2Apa, pORF2asApa	ORF2 inserted in sense or antisense orientation downstream of *GFP* by using the restriction enzyme *Apa* I.
pLacZ, pLacZas	*LacZ* inserted in sense or antisense orientation downstream of *GFP*.
p280-1*8, p280-2*8, p280-4*8, p280-5*8, p280-7*8, p280-8*8, p280-9*8	Eight copies of fragments 280-1, 280-2, 280-4, 280-5, 280-7, 280-8 and 280-9 inserted in sense orientation downstream of *GFP*.
pAlu*1, pAlu*2, pAlu*4, pAlu*8 , pAlu*14	One, 2, 4, 8 or 14 copies of *Alu* inserted in sense orientation downstream of *GFP*.
p280-1*8as, p280-2*8as, p280-4*8as, p280-5*8as, p280-7*8as, p280-8*8as, p280-9*8as	Eight copies of fragments 280-1, 280-2, 280-4, 280-5, 280-7, 280-8, 280-9 inserted in antisense orientation downstream of *GFP*.
pAlu*8as, pAlu*14as	Eight or 14 copies of *Alu* inserted in antisense orientation downstream of *GFP*.
p(AAACAAA)Rep, p(AAACAAA)Repas	AAACAAA simple repeat (736 bp) inserted in sense or antisense orientation downstream of *GFP*.
p(AG)Rep, p(AG)Repas	AG simple repeat (736 bp) inserted in sense or antisense orientation downstream of *GFP*.
p280-1~8as	280-1~8 fragment inserted in antisense orientation downstream of *GFP*.
p280-1*14, p280-1*14as	Fourteen copies of fragment 280-1 inserted in sense or antisense orientation downstream of *GFP*.
p280-4*1,p280-4*2, p280-4*4, p280-4*14	One, 2, 4, or 14 copies of fragment 280-4 inserted in sense orientation downstream of *GFP*.

**Table 2:** Primers and oligonucleotides used in this study.

Amplified fragments	Restriction enzyme	Sequence of primers (The underlined sequences refer to the restriction sites)
ORF2 (3825 bp)	*Xho* I / *Pst* I	Forward: 5'-ATCGCTCGAGCTTAAATGACAGGATCAAA TTCACAC-3' ; Reverse: 5'-ATCGCTGCAGTCAATTCCCACCTAT TAGGG-3'
ORF2as (3825 bp)	*Pst* I / *Xho* I	Forward: 5'-ATCGCTCGAGCTTAATCAATTCCCACCTAT TAGGG-3'; Reverse: 5'-ATCGCTGCAGATGACAGGATCAAATT CACAC-3'
ORF2Apa (3825 bp)	*Apa* I	Forward: 5'-ATCGGGGCCCCTTAAATGACAGGATCAA ATTCACAC-3'; Reverse: 5'-ATCGGGGCCCCTTAATCAATTCCCAC CTATTAGGG-3'
LacZ (3825 bp)	*Xho* I / *Pst* I	Forward: 5'-ATCGCTCGAGCTTAATGACCATGATTACG GATTCACTGG-3'; Reverse: 5'-ATCGCTGCAGGGAAACGCCAATAAC ATACAGTGAC-3'
LacZas (3825 bp)	*Xho* I / *Pst* I	Forward: 5'-ATCGCTCGAGCTTAGGAAACGCCAATAA CATACAGTGAC-3'; Reverse: 5'-ATCGCTGCAGATGACCATGATTACGG ATTCACTGG-3'
*Alu* (283 bp)	*Eco*R I / *Xba* I *Kpn* I / *Nhe* I	Forward: 5'-ATCGGAATTCTTAATCTAGATAAGGCT GGGCGCGGTGGCTCAC -3'; Reverse:5'-ATCGGGTACCATGCTAGCTGAGACGGA GTCTCGCTGTG-3'
280-1 (The first 280 bp of ORF2, from 1-280 bp)	*Eco*R I / *Xba* I *Kpn* I / *Nhe* I	Forward: 5'-ATCGGAATTCTTAATCTAGATAAATGA CAGGATCAAATTCACA-3'; Reverse: 5'-ATCGGGTACCATGCTAGCCTTTGTCTCTTT TGATCTTT-3
280-2 (The second 280 bp of ORF2, from 281-560 bp)	*Eco*R I / *Xba* I *Kpn* I / *Nhe* I	Forward: 5'-ATCGGAATTCTTAATCTAGATAAAAGG CCATTACATAATGGT-3'; Reverse: 5'-ATCGGGTACCATGCTAGCTTGGGGTGA AGAGTTCTGT-3'
280-4 (The fourth 280 bp of ORF2, from 1006-1285 bp)	*Eco*R I / *Xba* I *Kpn* I / *Nhe* I	Forward: 5'-ATCGGAATTCTTAATCTAGATAAAGAA GGCAAGAAATAACT-3'; Reverse: 5'-ATCGGGTACCATGCTAGCTTTCTCCTA GATTTTCTAG-3'
280-5 (The fifth 280 bp of ORF2, from 1675-1954 bp)	*Eco*R I / *Xba* I *Kpn* I / *Nhe* I	Forward: 5'-ATCGGAATTCTTAATCTAGATAAATCC ACCATGATCAAGTG-3'; Reverse: 5'-ATCGGGTACCATGCTAGCGGGAATGCT TCCGTTTTT-3'
280-7 (The seventh 280 bp of ORF2, from 2406-2685 bp)	*Eco*R I / *Xba* I *Kpn* I / *Nhe* I	Forward: 5'-ATCGGAATTCTTAATCTAGATAACCAT GCTCATGGGTAGG-3'; Reverse: 5'-ATCGGGTACCATGCTAGCTATCTCTGT TTTAGTACCAGTAC-3'
280-8 (The eighth 280 bp of ORF2, from 2933-3212 bp)	*Eco*R I / *Xba* I *Kpn* I / *Nhe* I	Forward: 5'-ATCGGAATTCTTAATCTAGATAAGGAA AACCTAGGCATTAC-3'; Reverse: 5'-ATCGGGTACCATGCTAGCCCACTTTTT GATGGGGT-3'
280-9 (The ninth 280 bp of ORF2, from 3213-3492 bp)	*Eco*R I / *Xba* I *Kpn* I / *Nhe* I	Forward: 5'-ATCGGAATTCTTAATCTAGATAAGTGA AGGACATGAACAG-3'; Reverse: 5'-ATCGGGTACCATGCTAGCTCCTAGATC CCTGAGGAAT-3'
AAACAAA oligonucleotide (78 bp)	*Eco*R I / *Xba* I / *Nhe* I / *Kpn* I	Template: 5'-ATCGGAATTCTTAATCTAGAAAACAAA AAACAAAAAACAAAAAACAAAAAACAAAAAACA GCTAGCATGGTACCCGAT-3'; Forward: 5'- ATCGGAATTCTTAATCTAGA-3'; Reverse: 5'-ATCGGGTACCATGCTAGC-3'
AG oligonucleotide (78 bp)	*Eco*R I / *Xba* I/ *Nhe* I / *Kpn* I	5' -ATCGGAATTCTTAATCTAGAAGAGAGAGAGAGA GAGAGAGAGAGAGAGAGAGAGAGAGAGGCTAGC ATGGTACCCGAT-3' Forward: 5'- ATCGGAATTCTTAATCTAGA-3'; Reverse: 5'-ATCGGGTACCATGCTAGC-3'
*GFP* probe (81 bp)		Forward: 5'-GGGCGAGGGCGATG-3'; Reverse: 5' -GTGGGCCAGGGCAC-3'
Neo probe (170 bp)		Forward: 5' -GCTCCTGCCGAGAAAGTATCC-3'; Reverse: 5'- CCCTGATGCTCTTCGTCCAGAT-3'

**Table 3:** Base content of 280-bp ORF2 fragments.

	Base number (%^1^) of ORF2 fragments						
	280-1	280-2	280-4	280-5	280-7	280-8	280-9
A	121 (44)	109 (39)	136 (49)	114 (41)	107 (38)	126 (45)	106 (38)
C	54 (19)	67 (24)	51 (18)	58 (21)	63 (23)	60 (21)	54 (19)
G	50 (18)	45 (16)	48 (17)	43 (15)	48 (17)	46 (16)	61 (22)
T	55 (20)	59 (21)	45 (16)	65 (23)	62 (22)	48 (17)	59 (21)

^1^Percentage in each 280-bp fragment.

## References

[Abrusanetal2008] Abrusán G., Krambeck H.J., Junier T., Giordano J., Warburton P.E. (2008). Biased distributions and decay of long interspersed nuclear elements in the chicken genome. Genetics.

[Allenetal2003] Allen E., Horvath S., Tong F. (2003). High concentrations of long interspersed nuclear element sequence distinguish monoallelically expressed genes. Proc Natl Acad Sci USA.

[Athanikaretal2004] Athanikar J.N., Badge R.M., Moran J.V. (2004). A YY1-binding site is required for accurate human LINE-1 transcription initiation. Nucleic Acids Res.

[Baileyetal2000] Bailey J.A., Carrel L., Chakravarti A. (2000). Molecular evidence for a relationship between LINE-1 elements and X chromosome inactivation: The Lyon repeat hypothesis. Proc Natl Acad Sci USA.

[Belancioetal2006] Belancio V.P., Hedges D.J., Deininger P. (2006). LINE-1 RNA splicing and influences on mammalian gene expression. Nucleic Acids Res.

[Boissinotetal2000] Boissinot S., Chevret P., Furano A.V. (2000). L1 (LINE-1) retrotransposon evolution and amplification in recent human history. Mol Biol Evol.

[BurwinkelandKilimann1998] Burwinkel B., Kilimann M.W. (1998). Unequal homologous recombination between LINE-1 elements as a mutational mechanism in human genetic disease. J Mol Biol.

[Costetal2002] Cost G.J., Feng Q., Jacquier A., Boeke J. (2002). Human L1 element target-primed reverse transcription *in vitro*. EMBO J.

[Deiningeretal2003] Deininger P.L., Moran J.V., Batzer M.A., Kazazian H.H. (2003). Mobile elements and mammalian genome evolution. Curr Opin Genet Dev.

[Dewannieuxetal2003] Dewannieux M., Esnault C., Heidmann T. (2003). LINE-mediated retrotransposition of marked Alu sequences. Nat Genet.

[Ergunetal2004] Ergün S., Buschmann C., Heukeshoven J., Dammann K., Schnieders F., Lauke H., Chalajour F., Kilic N., Strätling W.H., Schumann G.G. (2004). Cell type-specific expression of LINE-1 open reading frames 1 and 2 in fetal and adult human tissues. J Biol Chem.

[Fengetal1996] Feng Q., Moran J.V., Kazazian H.H., Boeke J.D. (1996). Human L1 retrotransposon encodes a conserved endonuclease required for retrotransposition. Cell.

[GrahamandBoissinot2006] Graham T., Boissinot S. (2006). The genomic distribution of L1 elements: The role of insertion bias and natural selection. J Biomed Biotechnol.

[Hackenbergetal2005] Hackenberg M., Bernaola-Galvan P., Carpena P., Oliver J.L. (2005). The biased distribution of alus in human isochores might be driven by recombination. J Mol Evol.

[Hanetal2004] Han J.S., Szak S.T., Boeke J.D. (2004). Transcriptional disruption by the L1 retrotransposon and implications for mammalian transcriptomes. Nature.

[HedgesandBatzer2005] Hedges D.J., Batzer M.A. (2005). From the margins of the genome: Mobile elements shape primate evolution. Bioessays.

[KazazianJrandMoran1998] Kazazian H.H., Moran J.V. (1998). The impact of L1 retrotransposons on the human genome. Nat Genet.

[Landeretal2001] Lander E.S., Linton L.M., Birren B., Nusbaum C., Zody M.C., Baldwin J., Devon K., Dewar K., Dovle M., FitzHugh W. (2001). Initial sequencing and analysis of the human genome. Nature.

[Lyon2000] Lyon M.F. (2000). LINE-1 elements and X chromosome inactivation: A function for “junk” DNA?. Proc Natl Acad Sci USA.

[MartinandBranciforte1993] Martin S.L., Branciforte D. (1993). Synchronous expression of LINE-1 RNA and protein in mouse embryonal carcinoma cells. Mol Cell Biol.

[MartinandBushman2001] Martin S.L., Bushman F.D. (2001). Nucleic acid chaperone activity of the ORF1 protein from the mouse LINE-1 retrotransposon. Mol Cell Biol.

[Martinetal2005] Martin S.L., Cruceanu M., Branciforte D., Wai-Lun Li P., Kwok S.C., Hodges R.S., Williams M.C. (2005). LINE-1 retrotransposition requires the nucleic acid chaperone activity of the ORF1 protein. J Mol Biol.

[Medstrandetal2002] Medstrand P., van de Lagemeat L.N., Mager D.L. (2002). Retroelement distributions in the human genome: Variations associated with age and proximity to genes. Genome Res.

[Moranetal1996] Moran J.V., Holmes S.E., Naas T.P., DeBerardinis R.J., Boeke J.D., Kazazian H.H. (1996). High frequency retrotransposition in cultured mammalian cells. Cell.

[Muotrietal2005] Muotri A., Chu V.T., Marchetto M.C.N., Deng W., Moran J.V., Gage F.H. (2005). Somatic mosaicism in neuronal precursor cells mediated by L1 retrotransposition. Nature.

[Okanoetal2008] Okano K., Zhang Q., Kimura S., Nartta J., Tanaka T., Fukuda H., Kondo A. (2008). System using tandem repeats of the cA peptidoglycan-binding domain from *Lactococcus lactis* for display of both N- and C-terminal fusions on cell surfaces of lactic acid bacteria. Appl Environ Microbiol.

[Pavliceketal2001] Pavlicek A., Jabbari K., Paces J., Paces V., Hejnar J., Bernardi G. (2001). Similar integration but different stability of Alus and LINEs in the human genome. Gene.

[Sassamanetal1997] Sassaman D.M., Dombroski B.A., Moran J.V., Kimberland M.L., Naas T.P., DeBerardinis R.J., Gabriel A., Swergold G.D., Kazazian H.H. (1997). Many human L1 elements are capable of retrotransposition. Nat Genet.

[Sciamannaetal2005] Sciamanna I., Landriscina M., Pittoggi C., Quirino M., Mearelli C., Beraldi R., Mattei E., Serafino A., Cassano A., Sinibaldi-Vallebona P. (2005). Inhibition of endogenous reverse transcriptase antagonizes human tumor growth. Oncogene.

[Sheenetal2000] Sheen F.M., Sherry S.T., Risch G.M., Robichaux M., Nasidze I., Stoneking M., Batzer M.A., Swerqold G.D. (2000). Reading between the LINEs: Human genomic variation induced by LINE-1 retrotransposition. Genome Res.

[SkowronskiandSinger1986] Skowronski J., Singer M.F. (1986). The abundant LINE-1 family of repeated DNA sequences in mammals: Genes and pseudogenes. Cold Spring Harb Symp Quant Biol.

[Smit1999] Smit A.F. (1999). Interspersed repeats and other mementos of transposable elements in mammalian genomes. Curr Opin Genet Dev.

[Swergold1990] Swergold G.D. (1990). Identification, characterization, and cell specificity of a human LINE-1 promoter. Mol Cell Biol.

[Weichenriederetal2004] Weichenrieder O., Repanas K., Perrakis A. (2004). Crystal structure of the targeting endonuclease of the human LINE-1 retrotransposon. Structure.

[Yangetal2004] Yang S., Smit A.F., Schwartz S., Chiaromonte F., Roskin K.M., Haussler D., Miller W., Hardison R.C. (2004). Patterns of insertions and their covariation with substitutions in the rat, mouse, and human genomes. Genome Res.

